# Tuberculosis among the Jews

**Published:** 1902-02-08

**Authors:** 


### EDITOR'S LETTER-BOX.

**[Our Correspondents are reminded that prolixity is a great bar to publication, and that brevity of style and conciseness of statement greatly facilitate early insertion.]**

### TUBERCULOSIS AMONG THE JEWS.

"A Jewess" writes: I should like to ventilate an opinion I have formed concerning the comparative immunity of the Jew from tuberculosis. I have remarked that the Jew usually lives well in comparison with the Gentile of the same class. The poorest Jews spend a large proportion of their earnings on food, which is usually of a flesh-producing nature, owing to the amount of oil they use in cooking. No doubt their freedom from alcoholism is an important factor in the matter.

